# Guidelines and Recommendations for Developing Interactive eHealth Apps for Complex Messaging in Health Promotion

**DOI:** 10.2196/mhealth.4423

**Published:** 2016-02-09

**Authors:** Kayla Joanne Heffernan, Shanton Chang, Skye Tamara Maclean, Emma Teresa Callegari, Suzanne Marie Garland, Nicola Jane Reavley, George Andrew Varigos, John Dennis Wark

**Affiliations:** ^1^ Department of Computing and Information Systems Melbourne School of Engineering The University of Melbourne Parkville Australia; ^2^ Health and Biomedical Informatics Centre Melbourne Medical School The University of Melbourne Parkville Australia; ^3^ University of Melbourne Department of Medicine Royal Melbourne Hospital Parkville Australia; ^4^ Royal Women's Hospital Parkville Australia; ^5^ Centre for Mental Health Melbourne School of Population and Global Health The University of Melbourne Parkville Australia; ^6^ Department of Dermatology Royal Melbourne Hospital Parkville Australia; ^7^ Royal Melbourne Hospital Bone and Mineral Medicine Parkville Australia

**Keywords:** mhealth, complex messaging, vitamin D, eHealth smartphone apps, interactive

## Abstract

**Background:**

The now ubiquitous catchphrase, “There’s an app for that,” rings true owing to the growing number of mobile phone apps. In excess of 97,000 eHealth apps are available in major app stores. Yet the effectiveness of these apps varies greatly. While a minority of apps are developed grounded in theory and in conjunction with health care experts, the vast majority are not. This is concerning given the Hippocratic notion of “do no harm.” There is currently no unified formal theory for developing interactive eHealth apps, and development is especially difficult when complex messaging is required, such as in health promotion and prevention.

**Objective:**

This paper aims to provide insight into the creation of interactive eHealth apps for complex messaging, by leveraging the Safe-D case study, which involved complex messaging required to guide safe but sufficient UV exposure for vitamin D synthesis in users. We aim to create recommendations for developing interactive eHealth apps for complex messages based on the lessons learned during Safe-D app development.

**Methods:**

For this case study we developed an Apple and Android app, both named Safe-D, to safely improve vitamin D status in young women through encouraging safe ultraviolet radiation exposure. The app was developed through participatory action research involving medical and human computer interaction researchers, subject matter expert clinicians, external developers, and target users. The recommendations for development were created from analysis of the development process.

**Results:**

By working with clinicians and implementing disparate design examples from the literature, we developed the Safe-D app. From this development process, recommendations for developing interactive eHealth apps for complex messaging were created: (1) involve a multidisciplinary team in the development process, (2) manage complex messages to engage users, and (3) design for interactivity (tailor recommendations, remove barriers to use, design for simplicity).

**Conclusions:**

This research has provided principles for developing interactive eHealth apps for complex messaging as guidelines by aggregating existing design concepts and expanding these concepts and new learnings from our development process. A set of guidelines to develop interactive eHealth apps generally, and specifically those for complex messaging, was previously missing from the literature; this research has contributed these principles. Safe-D delivers complex messaging simply, to aid education, and explicitly, considering user safety.

## Introduction

In 2013, there were 97,000 eHealth apps in major app stores [1]. Comments in the literature support the notion that these kinds of apps may be beneficial to health [1-4]. Some eHealth experts and technology advocates have predicted that one day they will prescribe an app as they would a medication [5]. Despite the potential benefits of eHealth apps [6], especially in the case of complex messaging, most eHealth interventions are developed commercially, while health organizations have lagged behind in this field [7].

The effectiveness of these apps can vary if development does not draw on change theories [2], employ scientific foundations, or involve health care professionals [3]. To date, much has been done broadly on designing for behavioral change [2,8,9] and on app development [9,10]. However, the domain of eHealth app development is still broadly under-researched. From a health perspective, involvement from health care professionals is crucial for efficacy and rigor [4,6,11], and patient safety must be considered as these apps have the potential to cause harm [11]. The reality that health information needs are somewhat different from general interests [12] means that health-specific considerations are important for app development in this domain.

More recently, clinician involvement has occurred [13], but there is still limited research in this area to guide development of eHealth apps. The lack of published examples describing eHealth app development makes it difficult to determine an eHealth app development process that is rigorous, research-based, and involves health care professionals. This paper aims to formulate recommendations to guide development of interactive eHealth apps by drawing on the principles from the broad app development literature and the Safe-D case study. The development of the D-Safe app to prevent vitamin D deficiency was used as case study. The app is currently being evaluated in a randomized controlled trial (RCT), which is underway (ACTRN 12613000972729). While the app developed is input into a current RCT, this paper focuses on how the app was developed and recommendations learned from the development. The RCT protocol and results will be the subject of later publications. Similarly, our work on personalizing messages for young women is published elsewhere [14].

High-level exposure to ultraviolet (UV) radiation from the sun is a major risk factor for skin cancer, yet UV exposure is needed for vitamin D synthesis in the skin. In Australia, skin cancer awareness has risen considerably in recent years. At the same time, research has demonstrated low vitamin D levels in various population samples despite abundant natural UV radiation. On the whole, Australians have adopted the Cancer Council’s “slip-slop-slap” campaign message [15], thereby reducing sun exposure and potentially inhibiting vitamin D synthesis. In one study, 31% of 18-24 year-olds residing in Australia were vitamin D deficient [16]. Currently, the message to the public is complex and potentially confusing: sunlight is needed for vitamin D, yet agencies, including the Cancer Council, urge individuals to limit “exposure to reduce the risk of melanoma” [17]. Difficulty stems from the need to balance adequate and safe UV exposure to stimulate sufficient vitamin D synthesis, against the harmful levels that increase risk of melanoma and other skin cancers. There are many variables to consider and conflicting recommendations. This is what we refer to as complex messaging, as the recommendations for safe sun exposure depend on an individual’s personal attributes and changing environment conditions. Vitamin D deficiency is diagnosed and managed primarily through general medical practitioners (GPs). However, a recent survey [18] found that the advice patients received differed from expert recommendations.

In addition, only a few foods such as oily fish, sun-exposed mushrooms, and eggs contain appreciable amounts of vitamin D [19]. Even with margarine and some other foods in Australia being fortified with vitamin D [17], the average Australian diet alone is not enough to achieve sufficient vitamin D. Moreover, barriers exist to widespread supplementation [17], such as cost and non-compliance issues. Therefore, safe sun exposure remains a high priority, as UV radiation is the major source of vitamin D [19]. However, UV varies based on location, season and time of the day, cloud cover, and pollution. In addition to these environmental factors, vitamin D synthesis is affected by skin pigmentation. For example, darker skin requires longer exposure than fairer skin to synthesize the same amount of vitamin D [20]. Vitamin D from sunlight is dependent on exposed skin as the greater the area covered, the less there is available for synthesis. Use of sunscreen has similar impact, diminishing UV absorption, thus reducing vitamin D synthesis [20].

To address these diverse variables, we selected an app as the best medium to provide the complex messaging required to guide safe but sufficient UV exposure, as it affords the two-way information exchange required for interactive, tailored recommendations. Utilizing an interactive eHealth app enables portability to record sun exposure duration and frequency as it occurs. Hence, more reliable exposure measurements [5,21,22] can be captured than is possible from traditional diary-based interventions, potentially improving safety and accuracy. Interaction is especially important because static one-way interventions would not be able to provide recommendations based on individual and external factors at the time of exposure, as is required for user safety. Moreover, we found that many young women aged 16-25 years, the target audience for the study and Safe-D app, wanted information regarding required sun exposure (36/47, 77%) while over half (32/47, 68%) believed an app could help improve vitamin D levels and also identified a need for better information than their GP currently provided.

The Safe-D study is limited to English-reading females aged 16-25 years residing in the State of Victoria, Australia. Therefore, the Safe-D app is in English only and designed for this target audience. Safe-D, and thus this research, does not include social media integration during its evaluation. There is a need for this limitation to restrict the opportunity for participants to share information with non-app intervention participants during its evaluation, insuring RCT integrity. Further to these limitations, the app is available only on Apple and Android devices, using existing technologies of Global Positioning System (GPS) and readily available UV data from the Australian Radiation Protection and Nuclear Safety Agency (ARPANSA) and Bureau of Meteorology (BOM). The limitation to Apple and Android devices only was based on Australian Interactive Media Industry Association (AIMIA) results that 49% of Australians own a smartphone, predominantly Apple and Android devices [23]. As AIMIA results are disaggregated from gender and age, a Web survey was conducted to understand the young female smartphone landscape. Of the respondents, 98% (56/57) owned a smartphone, 95% of those comprising Apple and Android devices, reinforcing the decision to develop for these platforms only.

### Theoretical Background

Safe-D development conformed to eHealth, persuasion, and interactivity best practices. A literature review was conducted to align development with current literature, presented thematically in this section. The aim of Safe-D was to persuasively encourage UV exposure. Persuasive technologies are those to prompt behavior change and reinforce learning [9]. A variety of concepts in the literature [2-4,9,10,24-28] were considered in the development of the app, including persuasion through monitoring and tracking, simplicity, rewarding behaviors, gamification, tailored messages, and improving user experience (UX).

###  Monitoring and Tracking

Eysenbach [2] found that participants liked to monitor, track, and review records, and he established that “simply viewing records…could prompt healthier behavior.” Along similar lines, it has been found that the interactivity of tracking physical activity led to an increase in steps through incremental changes [27]. Therefore, young women tracking other quantifiable events may do the same. Interactive eHealth apps for complex messaging should enable exposure tracking and performance indicators.

###  Simplicity

Dennison et al [2] also uncovered competing participant desires for accurate records without frequent data entry. Where entry is complicated and time-consuming, users discontinued tracking [10,26]. The premise behind these findings is expressed by Fogg [3] who argued that barriers that could prevent desired behavior must be removed for the behavior to occur. Therefore, as much as interaction is important, it seems that data entry by the users also has to be quick, easy, and convenient, while accurate.

###  Rewarding Behavior

Rewarding positive behaviors, rather than punishing non-compliance, leads to continued engagement [2,8,26,29]. Consolvo and Landay [8] found negative reinforcement does not encourage behaviors, nor does lack of credit for compliance. Therefore, it was important to consider how to reward behaviors while not punishing non-compliance.

###  Gamification

Gamification is the use of game elements in non-game contexts to provoke competition [30]. Calvo and Peters [31] found that provision of information alone only motivates change in 10% of individuals and suggested that interactive gamification may increase motivation and intervention compliance, a strategy also suggested by Segerstahl et al [10]. Given convergent recommendations, interactive eHealth apps for complex messaging should consider gamification to encourage use and improve compliance.

###  Tailored Messages

It is well published that tailored messages are most effective at motivation [2,8,26,28]. While messages are vital for an eHealth intervention to aid learning, doing so excessively dissuades users [2]. The contexts of target users must be considered to deliver relevant messages at opportune times for desired behavioral change to occur [3]. Clinicians also should be involved in complex message construction and messages tested for suitability and relevance with target users.

###  User Experience

UX encompasses “reactions and feelings, which arise from interactions with a system” [10]. UX elicitation can be accomplished by applying visually appealing designs [8,28]; a competent look and feel [28]; creative, fun, and humorous elements [26]; ease of use; error tolerance; and system responsiveness [9]. The literature indicates that for successful persuasion, UX must be considered [2,9,10]. Therefore, interactive eHealth app development should focus on user-centered design by involving users and leveraging researcher expertise in UX and human computer interaction.

## Methods

This research had dual goals of developing Safe-D while utilizing the process to understand development principles for interactive eHealth apps for complex messaging. These dual goals contribute to the development of a tangible solution in the form of the Safe-D app, while corresponding with intended outcomes of participatory action research [32,33]. Participatory action research was employed as it enabled a team of subject matter experts to work collaboratively with target users to develop the app, while researchers in the team reflected on the experience to garner recommendations. The experts involved included subject matter expert clinicians, a biostatistician, information systems researchers, external developers, public health practitioners, and target users.

### App Development

As is commonplace in qualitative research, development began relatively theory-free [34] in “round-table”-style discussions and ideation meetings with the team. Initial meetings were then used to fully appreciate and articulate the complex and confounding factors of achieving adequate vitamin D synthesis, which the app would have to cater for. These discussions were important to establish the range of variables to be considered, the diverse contexts of the target users, and, just as importantly, common understanding between the different groups of experts at the table. This was not only an important part of the app development process but was also the beginning of the participatory action research process.

A preliminary survey was conducted to further understand what young women wanted in an app, to inform design decisions. The survey was conducted online and advertised via a Melbourne Health media release, the “Netball Scoop” forum, VGen Core group, the Youth Affairs Council of Victoria monthly e-newsletter, and an email to past participants in the Young Female Health Initiative (a collaborative comprehensive health study of 16-25 year-old women in Victoria, Australia). A total of 71 respondents commenced the survey. Of these, 57 passed all eligibility criteria to participate, that is, being English-speaking females aged 18-25.

Developers were hired, and development followed stages from the software development lifecycle, selected due to its applicability to both business and health care scenarios [35]. The stages of our software development lifecycle were iterative and tailored to incorporate agile development ideologies [36]. These are described in Table 1. An existing algorithm [37] was selected to calculate recommendations for UV exposure due to applicability in New Zealand, similar in latitude to Australia.

**Table 1:** Development phases.

Development phase	Process and data generated to help develop the Safe-D App
Requirements analysis	Requirements were written and a literature review and focus groups were conducted.
Design	Wireframes were created to elicit non-functional and evolving requirements.
Design validation	Designs were validated in informal focus groups, an advisory private Facebook group, and informal “guerilla testing,” asking young women approached in public places for opinions regarding designs. Outcomes were used to (1) determine preferences, (2) validate designs as flexible, non-offensive, and culturally sensitive, (3) validate messages conveyed the intended message, and (4) test the app concept.
Development	The contracted developers created Safe-D for Apple and Android platforms iteratively and collaboratively with the other researchers.
Beta testing	Core team members who used with Apple and Android devices were given access to Safe-D. Additional beta testers were seconded from the larger team to ensure a range of platform and operating system configurations, making a total of 24 beta testers, consisting of 6 from the target demographic while others acted as expert reviewers.
Retrospective	This is the final phase, adopted from agile methodologies. Retrospectives are specific meetings reflecting on the development and identifying improvements. Retrospective data enabled triangulation so findings did not rely solely on observations of the authors.

Throughout each stage, available recommendations from the literature (as previously outlined) were used to help create the best experience in the app.

### Developing Recommendations

Researcher reflections and observational notes were used to highlight recommendations for future developments. This analysis was triangulated through data collected during retrospectives. Each member (ie, information systems and medical researchers, medical professionals, beta testers and app developers) shared what they thought went well, what did not, and what they would do differently next time based on this experience. Post-development, retrospective data were organized thematically through affinity mapping to check against initial researcher observations. This process condensed the data in a manageable format to identify any themes and relationships between themes, using an inductive approach, as per Neuman [33]. The data analysis enabled theoretical abstractions and pattern detection for identification of overarching principles for development of interactive eHealth apps for complex messaging.

## Results

As this research had the dual goals of first developing the Safe-D app and then using this process to develop recommendations for future app development, the results will be presented separately: the Safe-D app and development recommendations.

### Safe-D App

Safe-D aims to safely improve vitamin D status, through individualized UV exposure recommendations, messages, and learning. Safe-D will be presented by the major actions that users take within the app, which are initial setup, tracking UV exposure, monitoring progress, recording missed exposure, and viewing messages.

### Initial Setup

Initial app setup is required when the user accesses the app for the first time. Safe-D prompts for (1) push notification and GPS permissions needed to provide interactive personalized recommendations based on real-time locational UV, (2) answers to the Fitzpatrick skin-type questionnaire [38] to determine skin type (avoiding self-selection error), which is input once, stored, and used in calculations, and (3) avatar skin, hair, and eye color and hairstyle configuration. These selections are used to present an avatar that will remind the user of themselves, without tying skin tone to skin type, thus increasing attention and persuasion [3,9,10,25,26,39]. The avatar configurations enable users to comfortably represent themselves for greater persuasion through imitation. There were extensive UX considerations regarding avatar and clothing options. The avatar does not propagate unrealistic body image, and cultural awareness was considered through inclusion of the hijab and niqab options.

Tunneling, the practice of guiding users through 1 question per screen, was implemented to reduce cognitive load. All data entry is simple to encourage usage, and logon details can be remembered for automatic sign-in to remove barriers to future use. As Safe-D is intended for initial evaluation in an RCT, access must be restricted to only participants through password protection.

### Tracking UV Exposure

To record UV exposure, the avatar must first be dressed. This is a seamless and fun interaction used to record exposed skin and sunscreen protection. Sunscreen and clothing are required for exposure calculations. Therefore, they are explicitly prompted. Exposed skin entry is simple: the avatar enables easy, visual, touch entry of clothing to calculate exposed skin. For usability, the avatar defaults to clothing from the last exposure, removing a step if exposed skin remains stable exposure-to-exposure.

These inputs, with locational UV levels and skin type, are used to calculate and display safe exposure recommendations. As exposure has a safe limit, the timer cannot simply count down. Exposure counts down to zero, then up in red to indicate, alert to, and dissuade overexposure. Gamification promotes usage, inspired by Fish n’ Steps metaphor [30]: a fish whose health improved or deteriorated with exercise. In the study, steps did not increase in the prestudy pedometer-only phase, yet when using Fish n’ Steps there was a positive impact on 74% (14/19) of participants [30]. In Safe-D, a sunflower was selected to reiterate connotations of sun exposure. As exposure reaches target, the sunflower health display grows healthier and happier. If healthy exposure is exceeded, health declines. When the user stops timing, they are provided with interactive feedback regarding whether more exposure is required or exposure has been met, or exceeded.

### Monitoring Progress

There are two ways to monitor progress on the app: viewing today’s exposure or overall exposure. Today’s exposure is visible on the homepage by assessing the sunflower’s health state. The sunflower health state can also be assessed while the timer is running. Overall progress is monitored via statistics, displaying the current consecutive days-of-use in the form of a streak, and longest consecutive use. Streak here is used in the sporting sense to refer to a running total of consecutive days when the app was accessed. To encourage reaching exposure, without exceeding the defined safe limit, Safe-D enables easy entry and viewing of exposure, making it apparent when the safe limit is exceeded. The progress display of how much exposure the user has had each day allows them to adjust behavior accordingly. This visual display takes into account that exposure can be too high, too low, or just right for the most number of users. Inspiration was garnered from discussions with other app designers [40] at the Melbourne Quantified Self group meetup, which also points to the concept of a “goldilocks” zone (the zone for just the right level of access to information for users, or an ideal level of desired behavior). In this case, the goldilocks zone refers to just the right level of exposure to sunlight for each user, which is integrated with a traffic light display system. Therefore, the “just right” level of sunlight exposure is denoted as green, “too high” is red to signal danger, and “too low” is yellow to indicate it is suboptimal without reprimanding the user. This “just right” goldilocks zone is dependent on the acceptable statistics based on the algorithm behind the app.

### Recording Missed Exposure

Missed exposure can occur for two reasons: a user may be unable to get sun or may forget to record it. When unable to get sun, the “I Can’t Get Sun Today” feature records the reason. If users forget to start or stop the timer, they can retrospectively enter or delete using “My Sun Exposure.” Users may be unable to get sun for a number of reasons, and this function handles this sensitively without punishing a user by forcing them to lose their in-app streak.

### Exposure Recommendations

Safe-D aims to safely improve long-term vitamin D status through UV exposure. Thus, to prevent indefinite app reliance, information is required for learning. The complex messaging occurs on a per scenario basis (see Table 2), and different messages are delivered in different ways. These messages are complex because, while the same scenarios trigger messages for all users, the content of these messages varies based on the user’s skin type, how much skin they have exposed and whether they are using sunscreen or not, the current UV based on their current location, and the exposure they have already had for the day. Messages can be delivered in one or multiple modalities: in-app messages to deliver point-of-interaction information, push notifications requiring action and garnering attention to trigger this action as per the Fogg Behavioral Model [3], and mail messages available for later retrieval. Table 2 shows the scenarios that trigger messages to send and how these messages are delivered.

**Table 2:** Safe-D messages and medium per scenario.

Scenario	In app	Push	Mail
Timer stopped without reaching exposure	Yes		
Exposure reached	Yes	Yes	
Exposure exceeded	Yes	Yes	
Exposure exceeded and timer not stopped	Yes	Yes	
Exposure exceeded and “Get Vitamin D” accessed when UV is still above low	Yes		
Exposure exceeded and “Get Vitamin D” accessed when UV is now low	Yes		
Too much sun without reaching exposure, via the “Too Much Sun” function	Yes	Yes	
Safe-D not used		Yes	
UV in current location changes during exposure	Yes	Yes	
Forecast UV is extreme or very low		Yes	Yes

From our preliminary survey, 64% (7/11) of respondents provided an answer for the acceptable number of notifications per day, reporting 1-2 notifications per day as acceptable. However, beta testing revealed that 1 per day when the user was not actively using Safe-D was annoying and could potentially inhibit use. Push notifications send to a user’s phone only under the following circumstances: the timer is running and/or behavior needs to occur, Safe-D was not used the previous day, the user recorded “Too Much Sun” yesterday, and an educational message sent once a week.

### Viewing Messages

Messages can be viewed either as push notifications or through in-app mail. Messages can be sent to groups to improve effectiveness rather than generic messages sent to all. Other messages are interactive, based on user inputs. Mail was designed in a way that enables tailored messages to be sent to defined groups. Hardcoded events are recorded in the Safe-D dashboard that researchers can access and identify individuals who may need special messages. An example of how this could be implemented is to look at users who continually flag that they cannot get sun today because they are “too busy.” A sensitively tailored message could then be sent to such a participant to encourage them to make time. In more extreme cases if a participant continuously exceeds app-defined safe exposure limits, it would be possible for the non-blinded researcher to identify and contact them directly for their safety.

Educational notifications are delivered weekly. These are designed to be fun, non-clinical, and short. Messages cannot build on previously received messages as participants can start Safe-D at different times. Clinical experts in bone health and dermatology first reviewed all written copy, and expert bodies, such as the Cancer Council, are referenced where appropriate. Messages are framed positively to avoid reprimanding users [8], and they correlate to the state of the sunflower to reinforce the metaphor and learning. Messages were created to be interesting and non-repetitive, cycling through different wording to avoid monotony due to repetition.

## Discussion

### Key Development Recommendations from the Safe-D Study

As previously indicated, the literature [2-4,9,10,26-29,31] suggested the following design concepts: persuasion through simple tracking, rewarding positive behaviors, not punishing non-compliance, considering message timing, including gamification, and designing UX. We extended these design concepts in the form of the following recommendations based on key learnings from the Safe-D project.

#### Recommendation 1: Involve a Multidisciplinary Team in the Development Process

As recommended in the literature [9,30,31], multidisciplinary teams are important in similar contexts. One of the key challenges for such a team is to ensure understanding of the complexities of the problem and the cultures within the different disciplines involved. It was important to ensure that team members were able to meet face to face as much as possible for discussions, clarifications, safety advice, and approvals. It is also crucial to get all the information systems researchers and clinicians in agreement on the scope and requirements of the app before involving the app developers.

It is important to note that despite all the preparation in our study, it was not always possible to ensure that all roles and messages were fully understood. For example, developers may want a decision quickly, not appreciating that clinician review and approval was required. Similarly, clinicians may have unrealistic technical expectations. It is important to work through these issues and also ensure that there is adequate time set aside for the complexities of working in the multidisciplinary team. This recommendation reflects Gold et al’s findings [41] regarding multidisciplinary teams in their development of eHealth intervention on social networking sites.

#### Recommendation 2: Managing Complex Messaging is Crucial to Engage Users

Conveying the complex and potentially confounding sun exposure factors to users was challenging. The recommendations from the study for managing the complex messaging were three-fold: (1) minimize user input requirements into the app to keep it relatively simple, (2) deliver safe personalized key messages to users when appropriate, and (3) manage the push messages from a central server.

First, the principle of minimizing input while making the complexity as invisible to the user was important. Safe-D achieved this by managing external factors in the background and only asking users for information that the app could not detect. The external UV level data sources (ARPANSA and BOM) were input into an algorithm, which was essential to calculate safe, personalized recommendations. Use of an algorithm enabled clear output based on individualized inputs, as is required for complex messaging. The mechanics of this algorithm, however, should not be apparent to users. The algorithm helped to determine which messages are most relevant and appropriate to individuals. If no algorithm exists, it is recommended that teams take the time to create one that provides the required output by taking into consideration all inputs and develop automated test coverage to run algorithms for complex messaging through all possible input combinations, especially when there is no existing algorithm available.

Second, it is the messages themselves that convey the complex information simply, with the use of gamification to further simplify understanding. These messages can be delivered in one or multiple modalities: in-app messages to deliver point-of-interaction information, push notifications when action is required, garnering attention to trigger the desired behavior as per Fogg [3], and mail messages with content available for later retrieval. When employing gamification, the metaphor used should tie back to the health problem to reinforce learning by leveraging a heuristic to prompt for behavior when not using the app.

This research has built on work by Fogg [3] and Oinas-Kukkonen and Harjumaa [9]. Push notifications follow Fogg’s Behavioral Model as “without an appropriate trigger, behavior will not occur” [3]. The timing of exposure-related notifications should be designed to be during use and to not occur at inopportune times [9]. Similarly, push notifications regarding the same event should occur no more than twice, as constant prompts can lead to annoyance [9].

Third, the health messages should be simplified and controlled from a central server. This is so that messages can be updated when needed and pushed to users when relevant, without requiring them to update the app. It is useful to enable the delivery of customized messages to user subsets from the central server. In addition, this allows for later retrieval of education messages to reinforce education. It is then also easier to track which user has received which messages to help avoid repetitiveness.

#### Recommendation 3: Design for Interactivity

By designing for interactivity, we applied concepts from the literature [2-4,9,10,24-28] to provide tailored recommendations [8-10], remove barriers to use [3,8-10], and achieve simplicity [2,9]. Through developing Safe-D, we extended these concepts to contribute specific implementations of them for eHealth apps that future developments can utilize.

##### Tailored Recommendations

While the literature posits that tailored messages have greater persuasion [9,28], information on how to tailor these messages in an eHealth context is lacking. Thus, we expanded this principle based on lessons learned in message construction and beta testing. To postulate, recommendations should be based on input from the individual user and relevant external information, and based on each user’s interactions with the app (accounting for start of use of app, frequency of interaction, and nature of the interaction).

##### Removing Barriers to Use

Dennison et al [2], Fogg [3], and Segerstahl et al [10] recommended that, for persuasion to occur, barriers to use must be removed. This research provides specific mechanisms to remove barriers, based on those experienced by beta testers. Removal of these barriers was addressed in design improvements through mechanisms to avoid repeat data entry, including facilitating quick entry of login details by enabling “Next” and “Go” keyboard options to move through fields with ease; remembering login details; storing static information (information that does not change with each interaction), rather than prompting for it; defaulting inputs to last used, rather than requiring re-entry; and touch-only input.

##### Simplicity

While the literature states that data entry must be simple to encourage use [2,10], strategies for simplicity are lacking. This research provides specific recommendations to achieve simplicity in an eHealth intervention. Simplicity can be achieved by working with target users to determine input options; providing touch-only entry of these closed-input options/choices, rather than free-text input; providing key information on the homepage for at-a-glance assessment; and push notifications to prompt for action, reducing cognitive burden.

These strategies simplified the interface to a few simple clicks, rather than extensive data entry. The push notifications reduced the need for users to remember recommendations and exposure time. As a result, the Safe-D app is easy to use, requiring minimal time to learn, and adopts an intuitive interface.

In summary, development followed the concepts from the literature of iterative and flexible development, continuous evaluation and refinement, participatory development, a multidisciplinary team and involvement of end-users, and active participation to fully appreciate and understand the issue up front [9,30,31]. Despite this adherence, challenges were still faced owing to complex messaging and differences in focus between clinicians and developers. The recommended development principles are designed to reduce challenges faced in the development of future interactive eHealth apps for complex messaging.

### Generalizability

The principles we have presented in this paper were created through drawing inferences from Safe-D development. Safe-D takes multiple inputs and provides individualized and varying messaging to aid learning for users to optimize ongoing cutaneous vitamin D synthesis; continued exposure is required indefinitely to maintain vitamin D status. These principles can be leveraged for development of eHealth apps with similar individualized and complex messaging requirements where a one-size-fits-all message would be harmful. Examples of some potential eHealth apps that would require complex messages are described in Table 3.

**Table 3:** Potential future eHealth apps with complex messaging needs.

eHealth app topic	Complex messaging
Allergy	Allergy management requires ongoing monitoring and actions. An app would require personalized messages to each individual as their allergies and combination of allergies would vary.
Diabetes, hyperglycemia, hypoglycemia, and insulin resistance	Disordered blood glucose requires complex messaging based on real-time individual readings to provide correct advice for the individual’s requirement at the time, be it to take insulin, glucose, or maintain current levels.
Management of chronic illness	Many chronic conditions require individualized care plans based on the severity of the condition and any comorbidities. Care plan apps need to be able to accept multiple metrics that the user needs to track and deliver the right messages based on how they are tracking against each metric.
Musculoskeletal disorders	The recommendations such an app would need to provide on the amount and type of physical activity would need to take in to account confounding factors of disorder (eg, osteoporosis), age, flexibility, and any temporary injuries.

Potentially most of these conditions will require individualized and complex messaging with the move towards personalized medicine [42,43]. While these principles will aid in development of other eHealth and interactive apps, they will not all be directly transferable to certain situations. For example, some condition characteristics or demographics may make it impractical to have patients as part of the study team. It is suggested here that these principles do not represent a set procedure, but rather recommended guidelines.

### Limitations

This paper has not presented a detailed description of the Safe-D app to maintain the integrity of the RCT. This information will be included in future reports after the RCT is completed. While Safe-D has been extensively beta tested, resulting in improvements, formal evaluation was not performed as part of this research. The RCT will evaluate whether Safe-D succeeds in its purpose to safely improve vitamin D status in young women and, therefore, the applicability of these principles to complex messaging. The proposed principles may not be applicable to all complex messaging and are not currently ranked in priority. Further, as the findings are based on a reflective analysis from participatory action research, it is possible that the guidelines may have inherent bias. This was mitigated somewhat through the triangulation of data, including using beta testers who were not involved directly in the app development. These limitations can be addressed through future research directions.

### Future Directions

Future research, as part of the Safe-D study, should include an evaluation of the usability and utility of the app. The greater study will confirm whether or not the app is effective in safely improving vitamin D status. For example, it is possible to reach exposure limits for the day, to stop sun damage, without synthesizing enough vitamin D when limited skin is exposed. The algorithm may be updated during the study, as well as other changes based on participant interviews during site-visits regarding experiences, to improve effectiveness. A future improvement to Safe-D may include indicating to the user whether they will likely get adequate vitamin D based on what they are wearing, enabling them to change clothing or alter sunscreen use to improve synthesis.

As this research provides recommended principles for developing interactive eHealth apps for complex messaging, the results are largely theoretical at this stage and require validation before their suitability can be entirely understood. The lack of evaluation is a limitation to these principles. While behavior change was experienced anecdotally among the core researchers and beta testers, the Safe-D study will validate the app and therefore the principles applied in its development. Furthermore, future work in the eHealth app area should leverage these principles to validate them.

### Conclusion

This research has provided insights into how to develop interactive eHealth apps for complex messaging. This outcome was achieved by drawing on examples from the literature. A set of guidelines to develop interactive eHealth apps generally, and specifically those for complex messaging, was previously missing from the literature. This research has contributed these principles. Safe-D delivers complex messaging to simplify education, explicitly considering user safety. Therefore, it showed a set of recommendations that might help develop a complex eHealth app that is simple for target users to engage with.

## Abbreviations

AIMIA: Australian Interactive Media Industry Association
ARPANSA: Australian Radiation Protection and Nuclear Safety Agency
BOM: Bureau of Meteorology
GP: general practitioner
GPS: Global Positioning System
RCT: randomized controlled trial
UV: ultraviolet radiation
UX: user experience
